# Human AlkB Homologue 5 Is a Nuclear 2-Oxoglutarate Dependent Oxygenase and a Direct Target of Hypoxia-Inducible Factor 1α (HIF-1α)

**DOI:** 10.1371/journal.pone.0016210

**Published:** 2011-01-14

**Authors:** Armin Thalhammer, Zuzana Bencokova, Rachel Poole, Christoph Loenarz, Julie Adam, Linda O'Flaherty, Johannes Schödel, David Mole, Konstantinos Giaslakiotis, Christopher J. Schofield, Ester M. Hammond, Peter J. Ratcliffe, Patrick J. Pollard

**Affiliations:** 1 Henry Wellcome Building for Molecular Physiology, University of Oxford, Oxford, United Kingdom; 2 Chemistry Research Laboratory and The Oxford Centre for Integrative Systems Biology, Department of Chemistry, University of Oxford, Oxford, United Kingdom; 3 Gray Institute for Radiation Oncology and Biology, University of Oxford, Oxford, United Kingdom; 4 Department of Cellular Pathology, John Radcliffe Hospital, Oxford, United Kingdom; Chinese University of Hong Kong, Hong Kong

## Abstract

Human 2-oxoglutarate oxygenases catalyse a range of biological oxidations including the demethylation of histone and nucleic acid substrates and the hydroxylation of proteins and small molecules. Some of these processes are centrally involved in regulation of cellular responses to hypoxia. The ALKBH proteins are a sub-family of 2OG oxygenases that are defined by homology to the *Escherichia coli* DNA-methylation repair enzyme AlkB. Here we report evidence that *ALKBH5* is probably unique amongst the *ALKBH* genes in being a direct transcriptional target of hypoxia inducible factor-1 (HIF-1) and is induced by hypoxia in a range of cell types. We show that purified recombinant ALKBH5 is a *bona fide* 2OG oxygenase that catalyses the decarboxylation of 2OG but appears to have different prime substrate requirements from those so far defined for other ALKBH family members. Our findings define a new class of HIF-transcriptional target gene and suggest that ALKBH5 may have a role in the regulation of cellular responses to hypoxia.

## Introduction

Recent work has revealed an extensive family of Fe(II) and 2-oxoglutarate (2OG) dependent oxygenases encoded in the human genome (reviewed in [1]). Some, for instance the HIF hydroxylases, are involved in the cellular response to hypoxia [2]; Other family members have diverse functions, including fatty acid metabolism, histone demethylation, and DNA repair [1], [3], [4], [5]. Human homologues of the *Escherichia coli* DNA repair enzyme AlkB are termed ALKBH enzymes. Some ALKBH enzymes have been demonstrated to function as nucleic acid demethylases, catalyzing the oxidative demethylation of 1-methyladenine and 3-methylcytosine in DNA and RNA [3], [6], [7]. Eight human AlkB homologues (ALKBH1-8) have been predicted, of which three (ALKBH1-3) have been shown to exhibit nucleic acid demethylation activity [8], [9], [10]. Additionally, a DNA lyase activity has been recently described for ALKBH1 that is Fe(II) and 2OG independent [11]. ALKBH8 expression has been implicated in bladder cancer progression. Recently, a tRNA methyltransferase activity of ALKBH8 has been described and implicated in translational decoding [12], [13], [14].

Several 2OG oxygenases, including two of the HIF hydroxylases themselves, and more recently, several of the JmjC domain-containing histone demethylases (JMJDs), have been identified as hypoxia-inducible gene products [15], [16], [17], [18], [19], [20], [21], [22], [23].

In order to test whether human 2OG oxygenases acting, or potentially acting, on nucleic acids are regulated by hypoxia, we compared the expression of *ALBKH* genes in normoxic and hypoxic conditions and identified *ALKBH5* to be induced by hypoxia. Analysis of the *ALKBH5* promoter *in silico* identified two putative HIF binding sites. Through chromatin precipitation studies we demonstrate that one of these sites binds HIF-1α. Analysis of cell lines bearing genetic interventions on the HIF pathway, confirm that *ALKBH5* is a novel HIF-1α responsive gene.

## Results and Discussion

### *ALKBH5* is a hypoxia-induced gene

**Figure 1:**
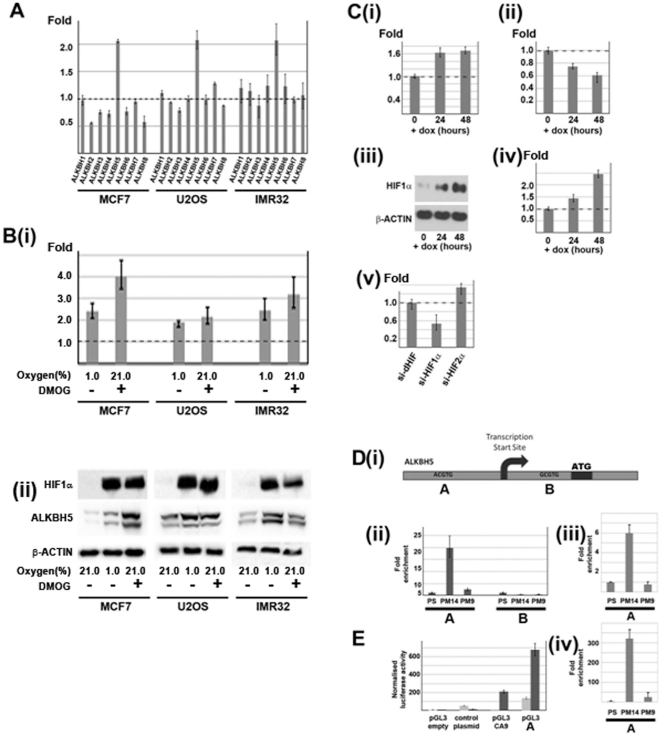
Hypoxia upregulates ALKBH5 *via* HIF-1α. (**A**) Hypoxic induction of *ALKBH1-8* in MCF7, U2OS and IMR32 cells. Values are the fold increase in mRNA in cells cultured for 16 hours in hypoxia (1.0% oxygen) relative to parallel cultures in normoxia (21% oxygen). Only *ALKBH5* is significantly upregulated (p<0.01) in each cell type. (**B**)(**i**) *ALKBH5* mRNA was significantly induced in MCF7, U2OS and IMR32 cells by hypoxia (1.0% oxygen for 16 hours) and DMOG (1 mM for 16 hours). (**ii**) Immunoblot demonstrating increased protein levels of HIF-1α (top panel) and ALKBH5 (middle panel) under hypoxia and DMOG treatment. Protein loading is indicated by β-actin signal (bottom panel). (**C**) Genetic manipulation of the HIF/VHL system in normoxia modulates *ALKBH5* expression. Inducible HIF-1α overexpression in U2OS cells and subsequent RT-Q-PCR analysis (**i**) shows a significant increase in *ALKBH5* mRNA after doxycycline administration. Expression of *VHL* in RCC4 cells and subsequent RT-Q-PCR analysis (**ii**) shows significant decrease in *ALKBH5* mRNA by 48 hours after doxycycline administration. Inducible peptide blockade of HIF-1α degradation demonstrating increased expression of HIF-1α protein (**iii**) at 24 hours (lane 2) and 48 hours (lane 3). RT-Q-PCR analysis (**iv**) shows a significant (p<0.01) increase in *ALKBH5* mRNA after doxycycline administration. RT-Q-PCR analysis (**v**) demonstrates significant reduction in hypoxic induction of *ALKBH5* mRNA by HIF-1α directed siRNA. These changes were not observed in siRNA directed against *Drosophila* HIFα or human HIF-2α. Knockdown for each HIFα subunit was greater than 90% as confirmed by both RT-Q-PCR and immunoblotting (data not shown). (**D**) HIF-1α binds the −482 to −486 promoter region of the *ALKBH5* gene and mediates hypoxic induction of a luciferase reporter in MCF7 cells. (**i**) Figure indicating 2 putative HREs (A,B) and their relative positions to the transcriptional and translational start sites. (**ii**) DNA encompassing HRE A was significantly enriched in chromatin precipitated with PM14 (anti-HIF-1α) but not with pre-immune sera (PS) or PM9 (anti-HIF-2α) in both (**iii**) CAKI-1 and (**iv**) RCC4 renal cancer cells. No enrichment was observed in DNA encompassing HRE B. (**E**) MCF7 cells were transfected with pGL3promoter vectors expressing the putative *ALKBH5* HRE A sequence. pGL3promoter vectors that were either “empty” or contained the *CA9* HRE served as controls as did an “irrelevant” vector. Luciferase activities were normalized to those of β-galactosidase in each sample. Luciferase activity was significantly increased after 16 hours of DMOG (1 mM) treatment in cells expressing pGL3-CA9 and pGL3-ALKBH5[HRE A] but not in the control plasmid or “empty vector.

To determine the extent of regulation by hypoxia among the human *ALKBH* family, we exposed a series of cell lines to hypoxia and tested for alterations in transcript levels by RT-Q-PCR across all *ALKBH* family members. The results shown in Figure 1α reveal a consistent pattern of regulation by hypoxia. *ALKBH5* was consistently up regulated in response to hypoxia (>2-fold in MCF7, U2OS and IMR32 cells), whereas none of the other *ALKBH* genes showed significant changes in expression. To determine whether induction by hypoxia was mediated by HIF hydroxylase pathways we next compared the action of hypoxia with that of dimethyl oxalylglycine (DMOG), a cell penetrating, generic 2OG oxygenase inhibitor. The results for MCF7, U2OS and IMR32 cells (Figure 1Bi) and reveal very similar patterns of mRNA induction to those observed following exposure of cells to hypoxia and DMOG, for A*LKBH5*. In line with the observed increase in *ALKBH5* mRNA levels, immunoblotting of cell extracts revealed that ALKBH5 protein levels were also increased substantially, both in hypoxic conditions and after DMOG treatment (Figure 1Bii). To further explore the dependence of *ALKBH5* induction on the HIF system, we measured changes in *ALKBH5* mRNA in a series of cell lines in which we were able to manipulate HIF in normoxic conditions (Figure 1C). Doxycycline-mediated induction of HIF-1α, either directly in U2OS transfectants bearing an inducible HIF-1α transgene, or indirectly in U2OS transfectants expressing a peptide encompassing the HIF-1α C-terminal prolyl hydroxylation site that blocks degradation of the native HIF-1α molecule, resulted in sustained induction of *ALKBH5* mRNA (Figure 1Ci,iii,iv). Conversely, doxycycline-mediated re-expression of HA-tagged wild-type *VHL* transgene into VHL-defective RCC4 cells [24] reduced *ALKBH5* mRNA levels, in line with restoration of VHL-mediated degradation of HIF (Figure 1Cii). Finally, we tested the role of HIF-1α and HIF-2α isoforms using siRNA mediated knockdown in hypoxic MCF7 cells. As indicated in (Figure 1Cv) siRNA directed against HIF-1α substantially reduced expression of *ALKBH5* whereas siRNA directed against HIF-2α resulted in a modest, but statistically insignificant increase, of *ALKBH5* mRNA. These results indicate that induction of *ALKBH5* by hypoxia is mediated directly or indirectly by HIF-1α.

Bioinformatic analysis of the *ALKBH5* locus revealed two putative hypoxia-response elements (5′-RCGTG-3′) at −486/−482 (herein termed HRE A) and +265/+269 (herein termed HRE B), referenced to the transcriptional start site (Supplementary figure S1). Both HIF binding sites displayed high conservation across other vertebrate genomes, suggesting functional conservation (Supplementary figure S1). Therefore, we carried out both chromatin immunoprecipitation (ChIP) and luciferase reporter assays to test whether *ALKBH5* is a direct HIF transcriptional target gene (Figure 1D,E). DNA from MCF7 cells was precipitated using anti-HIF-1α antibodies (PM14) or anti-HIF-2α antibodies (PM9) or control, pre-immune sera; precipitated DNA was subject to RT-Q-PCR analysis using primers designed to amplify each of the predicted HREs. Significant and reproducible enrichment of DNA containing HRE A at the *ALKBH5* locus was observed with anti-HIF-1α (>18-fold), but not anti-HIF-2α antibodies (Figure 1Dii). In contrast, no enrichment was observed at HRE B with either anti-HIF-1α or anti-HIF-2α. The ability of HIF-1α to bind the *ALKBH5* promoter in different cell lines was confirmed by ChIP in the renal cancer cell lines CAKI (Figure 1Diii) and RCC4 (Figure 1Div). Finally, a promoter fragment containing the *ALKBH5* HRE A was sufficient to drive hypoxia inducible expression of a luciferase reporter gene (Figure 1E) in HeLa cells at a comparable level to that seen with the promoter of a known HIF-target gene, carbonic anhydrase IX (*CAIX*) [25].

### ALKBH5 is a 2OG Oxygenase

**Figure 2:**
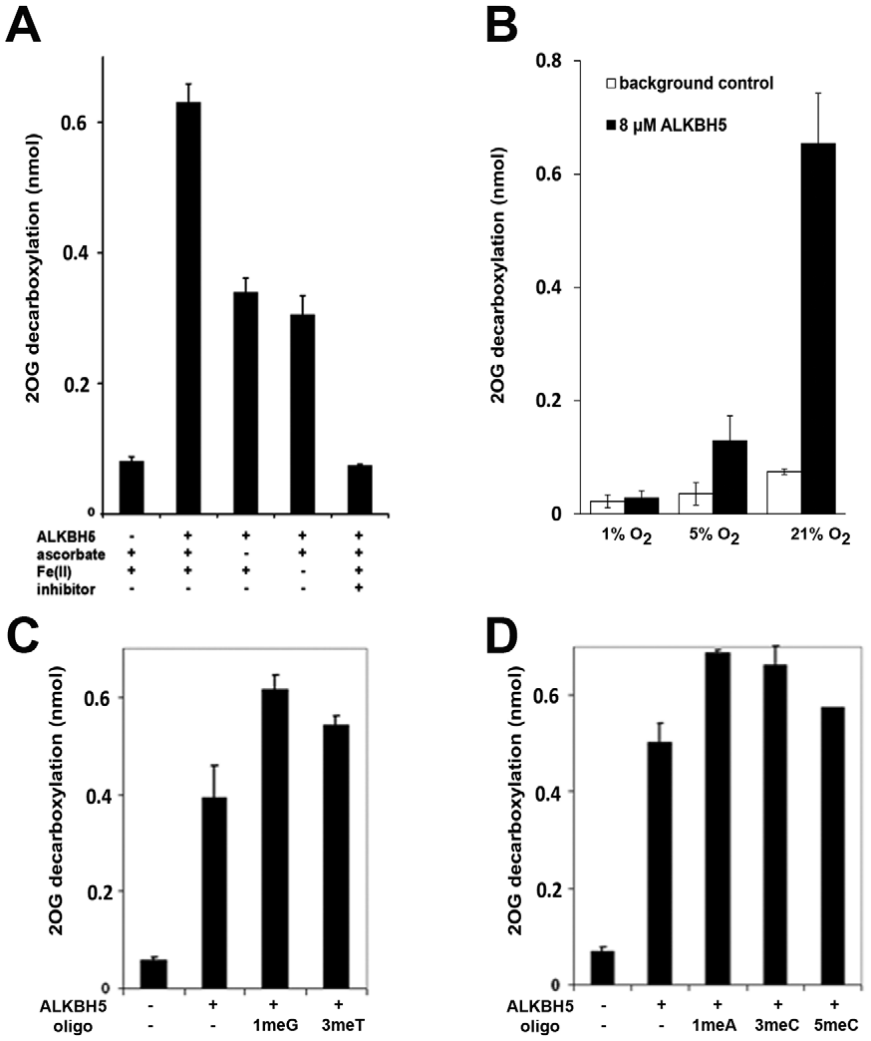
ALKBH5 displays biochemical characteristics of a functional 2OG-dependent oxygenase *in vitro*. (**A**) Enzyme-dependent stimulation of uncoupled turnover that was dependent on the cofactors ascorbate, and Fe(II) and inhibited by 1 mM of the generic 2OG oxygenase inhibitor pyridine-2,4-dicarboxylate (2,4-PDCA). (**B**) Enzyme-dependent stimulation of uncoupled 2OG turnover that was sensitive to the oxygen concentration. Turnover was significantly reduced under moderate hypoxia (5.0% oxygen) and negated under more severe hypoxia (1.0%) (**C**) Testing of potential oligonucleotide substrates; 1-methylguanine (1meG) 3-methylthymine (3meT) and (**D**) 1-methyladenine (1meA), 3-methylcytosine (3meC), 5-methylcytosine (5meC). No significant increase in ALKBH5 activity was observed in the presence of methylated oligonucleotides.

To investigate whether *ALKBH5* encodes an active 2OG oxygenase, we prepared purified recombinant ALKBH5 and tested the protein for catalytic activity in assays for 2OG decarboxylation. An *N*-terminal truncated protein, ALKBH5_66–292_, was produced in a soluble recombinant form in *E. coli* and was purified to near homogeneity, as assessed by SDS-PAGE analysis. Based on the knowledge that many 2OG oxygenases can oxidise 2OG to CO_2_ and succinate in the absence of their prime substrate, we employed an assay for ALKBH5 that measures the conversion of 1-[^14^C]-2OG to succinate and [^14^C]-CO_2_. These assays demonstrated that ALKBH5 stimulated 2OG decarboxylation in a manner that was dependent on supplementation with the 2OG oxygenase co-factors, Fe(II) and ascorbate, and inhibited by the 2OG oxygenase inhibitor pyridine-2,4-dicarboxylate (2,4-PDCA) thus indicating that ALKBH5 is an active 2OG oxygenase (Figure 2A). Increased transcription of the HIF prolyl hydroxylases (PHD2 and 3) is observed in hypoxia and has been proposed to compensate for the reduction in enzymatic activity and modulate hypoxia signaling [26]. Therefore, to further investigate the functional significance of hypoxic induction of ALKBH5, we analysed the effect of different oxygen levels (21%, 5.0% and 1.0%) on ALKBH5 activity (Figure 2B). Enzyme activity was reduced up to 75% at 5.0% oxygen and virtually absent at 1.0% oxygen (Figure 2B). It is therefore possible that upregulation of ALKBH5 in hypoxia compensates, at least in part, for the reduced enzymatic activity. Given that among ALKBH family members, induction by hypoxia appears to be strictly confined to ALKBH5 this might indicate that the enzyme has some specific function in hypoxic cells and it would be of interest to determine the correlation between ALKBH5 expression and oxygen levels in normal and neoplastic mammalian cells and tissues. To investigate whether ALKBH5 possesses DNA demethylase or DNA hydroxylase activity against known substrates for this class of enzymes, we prepared a set of 18-mer single-stranded oligonucleotides containing modified bases including 1-methyladenine (1meA), 3-methylcytosine (3meC), 5-methylcytosine (5meC), 1-methylguanine (1meG) and 3-methylthymine (3meT) by solid-phase oligonucleotide synthesis. However, none of these potential substrates significantly increased ALKBH5-mediated 2OG turnover and none were converted to demethylated or hydroxylated products, as indicated by LC-MS analysis (Figure 2C, D, Supplementary figure S2). Whilst we cannot rule out the possibility that full-length post-translationally modified ALKBH5 may act on these or other substrates, the observation that under *in vitro* conditions ALKBH5 does catalyze 2OG turnover but did not result in chemical modification of the tested potential substrates argues against this. With this caveat, our results suggest that either ALKBH5 does not act on these modifications, or only does so in a different context.

### Cellular localisation and potential role in DNA repair of ALKBH5

**Figure 3:**
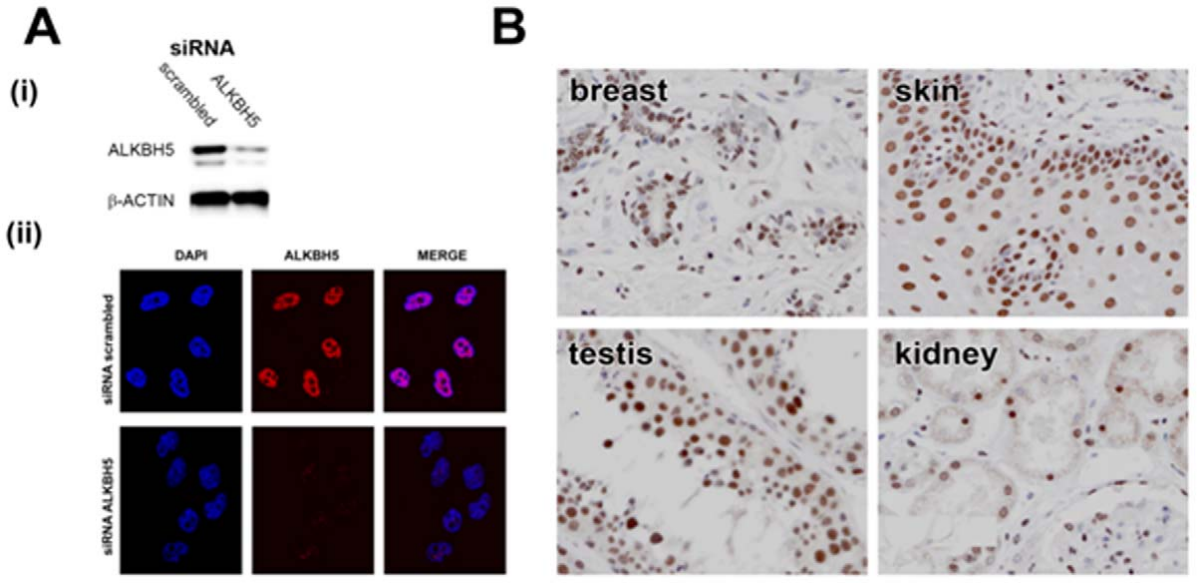
ALKBH5 localizes predominantly to the nucleus. (**A**)(**i**) Immunoblot demonstrating protein levels of ALKBH5. U2OS cells were treated with either a scrambled (control) siRNA sequence (left lane) or siRNA directed against *ALKBH5* (right lane). Protein loading is indicated by β-actin. (ii) Immunofluorescence-based detection of ALKBH5 protein in U2OS cells treated with either a scrambled (control) siRNA sequence (top panel) or siRNA directed against *ALKBH5* (bottom panel). (**B**) Nuclear ALKBH5 expression in normal breast, skin, testis and kidney. Original magnifications ×400.

To further investigate a potential role for ALKBH5 in nucleic acid metabolism, we characterised its cellular localization. Previously, *N*-terminally (GFP)-tagged ALKBH5 has been shown to localise throughout the cell [27]. Firstly, we verified that our antibody was specific to endogenous ALKBH5 and therefore sensitive to decreased levels of ALKBH5 in cultured cells. In keeping with this ALKBH5 immunoreactivity was substantially suppressed in U2OS cells treated with siRNA against ALKBH5 in comparison with that treated with a scrambled (control) siRNA (Figure 3A(i)). Next, U2OS cells were stained with anti-ALKBH5 (shown in red) and 4′,6-diamidino-2-phenylindole (DAPI) as a nuclear marker (shown in blue) (Figure 3A(ii)). We observed almost complete loss of nuclear staining when employing the siRNA directed against *ALKBH5* (Figure 3A(ii)). Furthermore, Taqman RT-Q-PCR analysis confirmed a >10-fold reduction of *ALKBH5* at the mRNA level but no changes in mRNA levels of *ALKBH1,-2,-3,-4, -6,-7 or -8* (data not shown). Our data shows that endogenous ALKBH5 resides predominantly in the nucleus with only sparse cytoplasmic staining. Immunohistochemical staining of human tissues (Figure 3B) indicates that ALKBH5 also localises primarily to the nucleus *in vivo*.

Severe hypoxia induces a DNA damage response which includes both ataxia telangiectasia and Rad3 related/ataxia telangiectasia mutated (ATR/ATM) activation [28], [29]. The hypoxia-induced DNA damage response is unusual in that DNA damage is not detected by either comet assay or tumor protein p53 binding protein 1 (53BP1) foci formation, but histone H2AX is phosphorylated (to form foci of γH2AX) [30]. More recently, depletion of dNTPs in these hypoxic conditions was shown to lead to the stalling of replication forks which in turn initiates a DNA damage response [31]. In contrast to hypoxia, reoxygenation has been shown to induce significant levels of ROS-dependent DNA damage [32]. Given that ALKBH5 is a HIF-induced nuclear protein with a possible role in DNA repair (based on homology to *E.coli* AlkB), we investigated a potential role for ALKBH5 in the context of DNA damage/repair under conditions of hypoxia/reoxygenation. Comparison of U2OS cells treated with ALKBH5 siRNA or a scrambled (control) siRNA allowed us to analyse the ALKB5-dependence of the response to hypoxia/reoxygenation by assessing γH2AX and 53BP1 foci formation (Supplementary Figure S3). siRNA knock downs were validated by RT-Q-PCR and were at least 90% efficient (data not shown). In each case the numbers of cells containing more than 10 nuclear foci were scored. The levels of γH2AX increased during hypoxia and persisted for at least 8 hours after reoxygenation. In contrast, as previously shown [30], 53BP1 did not form foci until reoxygenation. In each case the loss of ALKBH5 did not affect the response indicating that the components of the DNA damage response and repair are not dependent on ALKBH5 levels. We then extended these studies to consider if loss of ALKBH5 during hypoxia/reoxygenation affects cell survival by carrying out clonogenic survival assays (Supplementary Figure S3). Loss of ALKBH5 did not affect cell survival after hypoxia/reoxygenation indicating that hypoxia-induced ALKBH5 does not have an essential role in this process. As a final test, we carried out a second clonogenic survival experiment in moderate hypoxic (2% O_2_) conditions with the addition of γ-irradiation. The γ-irradiations were carried out under hypoxic conditions (2% O_2_) but cells were returned to normal culture conditions 30 minutes after irradiation (Supplementary ). Again, no effect of loss of ALKBH5 was determined. Together, these data indicate that hypoxia-induced ALKBH5 does not have a role to play in DNA repair or the DNA damage response.

### Conclusions

Taken together our studies identify ALKBH5 as a 2OG dependent oxygenase that is inducible by hypoxia through the action of HIF-1α. Induction of ALKBH5 by HIF-1α was unique amongst the known ALKBH family members suggesting a specific role for ALKBH5 in the response to hypoxia, as has recently been postulated for other 2OG dependent oxygenases that modulate patterns of histone methylation in hypoxic cells [20]. Though ALKBH5 is largely confined to the nucleus in human cancer cells we have not yet clearly defined its function. Its substrate(s) requirements also appear to be different from those so far defined for other ALKBH family members. Nevertheless our demonstration that recombinant ALKBH5 supports the uncoupled decarboxylation of 2OG in a manner that is co-factor and inhibitor dependent clearly assigns the protein as a functional 2OG oxygenase and should enable further physiological analysis.

## Materials and Methods

### Cell culture

Human osteosarcoma (U2OS), breast cancer (MCF7), hepatoma (HEP3B) and cervical carcinoma (HeLa) cells were cultured in Dulbecco's modified Eagle medium (DMEM) (Invitrogen) supplemented with 10% (v/v) fetal calf serum (FCS) (Sigma). Human neuroblastoma cells (IMR32) were cultured in Eagle's minimal essential medium (Invitrogen) with 10% (v/v) FCS, 2mM glutamine (Invitrogen) and non-essential amino acids. All cells were maintained in a humidified atmosphere of 5% CO_2_ and 21% O_2_. U2OS, HeLa, MCF7, HEP3B and IMR32 cells were obtained from Cancer Research UK. R38 (human renal carcinoma (RCC4) cells that express a tetracycline-inducible hemagglutinin (HA)-tagged von Hippel-Lindau (*VHL*) transgene), C29 (U2OS cells expressing tetracycline inducible HIF-1α) and myc19 (U2OS cells expressing a tetracycline inducible c-myc-tagged HIF-1α peptide (residues 549–582) have been previously described [24], [33] and were cultured in DMEM with 10% (v/v) tetracycline-free FCS (BD Bioscience) supplemented with 1 µg/ml G418 and 5 µg/ml Blasticidin (Sigma). In the experimental work, cells were cultured under normoxic conditions (5% CO_2_, 21% O_2_), in moderate hypoxia (16 hours at 5% CO_2_, 0.5–2% O_2_, Invivo_2_ hypoxia workstation), or severe hypoxia (16 hours at 0.02% O_2_, Bactron chamber, Shel Lab). Where specified, cells were treated with dimethyloxalyl glycine (DMOG) (1mM for 16 hours).

### siRNA Treatment of Cells and clonogenic survival assays

MCF7 and U2OS cells were seeded at 30% confluency and grown in normoxic conditions. Cells were transfected twice at 24 hour intervals with siRNA (20nM) using Oligofectamine (Invitrogen) according to the manufacturer's protocol. The siRNA oligonucleotide sequences used for HIF-1α and HIF-2α were as described [34]. For survival assays, U2OS cells were treated with siRNA as above, exposed to hypoxia (0.02% O_2_) for the times indicated, and then returned to normal culture conditions to form colonies (approx 10 days) which were then stained with crystal violet.

### RNA Extraction and cDNA Synthesis

Total RNA was extracted using the Sigma Total RNA kit (Sigma) according to the manufacturer's protocol. First strand cDNA synthesis was generated from 5 µg of total RNA (GE Healthcare).

### Protein Extraction and Immunoblot Analysis

Preparation of cell extracts and immunoblot analyses were performed as described [35]. Primary antibodies used were mouse anti-HIF-1α (BD Transduction Laboratories), rabbit anti-HIF-2α (Novus Biologicals), rabbit anti-ALKBH5 (Sigma), mouse horseradish peroxidase (HRP)-conjugated anti-HA (Dako), rabbit phospho-H2AX (Abcam), mouse anti-β-actin (Santa Cruz) and mouse HRP-conjugated anti-β-actin (Abcam).

### Immunofluorescence

Transiently transfected U2OS cells were fixed with 4% paraformaldehyde in phosphate-buffered saline (PBS) for 15 min, permeabilized in Triton X-100 (0.05% in PBS) and stained with anti-ALKBH5 (Sigma), anti-53BP1 (Novus Biologicals) or anti-γH2AX (Upstate). Secondary antibodies were anti-rabbit-alexa-fluor-594 or anti-mouse-alexa-fluor-594 (Invitrogen). Cells were counterstained with 4,6-diamino-2-phenylindole (DAPI) fluorescent dye (Vector Laboratories), as a nuclear marker.

### Immunohistochemistry

Immunohistochemistry was carried out as described [36] using the EnVision kit (Dako) and rabbit anti-ALKBH5 (Sigma).

### Chromatin Immunoprecipitation (ChIP) Assays

DNA-binding proteins were cross-linked to DNA using formaldehyde at a final concentration of 1% (w/v) for 10 minutes at 25°C, followed by treatment with glycine (125 mM) for a further 5 minutes. Cells were washed in PBS, lysed in SDS lysis buffer, and sonicated (Sonics & Materials, VCX 500). The supernatant was collected by centrifugation and pre-cleared with protein A agarose beads (Millipore). Chromatin was then incubated overnight with rabbit polyclonal anti-sera to HIF-1α (PM14) and HIF-2α (PM9) [37] before adding protein A agarose for a further hour. Pre-immune serum was used as a negative control. The beads were washed and immunoprecipitated complexes were eluted into 1% SDS, 0.1 M sodium bicarbonate elution buffer. Cross-linking was reversed by overnight incubation at 65°C and followed by protein digestion with proteinase K. DNA was recovered by phenol/chloroform extraction and ethanol precipitation.

### Real-time quantitative PCR

RT-Q-PCR for mRNA quantification employed Taqman gene expression assays on a StepOne thermocycler (Applied Biosystems). Normalization was to *HPRT1* mRNA and relative gene expression was calculated using the ΔΔCT method. A cDNA template of 10 ng per reaction was used and 3 biological replicates, each in triplicate, were performed for each experiment. For quantification of HIF binding sites, ChIP DNA (5 ng) from each of input, preimmune sera, PM14 and PM9 was subject to RT-Q-PCR using oligonucleotides designed to amplify putative HRE consensus sequences (Supplementary Figure S1). Fold enrichment was determined by the ΔΔCT method. Statistical comparisons were performed using Student's unpaired t-test.

### Luciferase Reporter Assays

Cells at 70% confluence in 6-well plates were transfected with the specified *ALKBH5* luciferase reporter plasmid (0.5 µg), together with pRSV β-galactosidase control plasmid (0.1 µg). Luciferase assays were performed 36 h post-transfection with a luminometer; relative luciferase light units were normalized to β-galactosidase activity.

### Expression plasmids and protein purification

For production in *E. coli*, a human *ALKBH5* cDNA encoding an *N*-terminal His_6_-tag linked to residues 66-292 was inserted into the pNIC28-Bsa4 expression vector. For large-scale ALKBH5 production, plasmids were transformed into *E. coli* Rosetta 2 (DE3) cells, induced with 0.5 mM IPTG at OD 0.6 and grown for 16 hours at 18°C. Cells were harvested and lysed by sonication in 20 mM Tris-HCl (pH 7.9), 0.5 M NaCl, 5 mM imidazole and 10 mM MgCl_2_ in the presence of DNAse and protease inhibitor cocktail (Roche Complete™ Mini EDTA-free); soluble protein was purified by immobilized Ni(II) affinity chromatography using Ni-NTA HisBind® resin (Novagen) and subsequent gel filtration chromatography. Protein of >95% purity (by SDS/PAGE analysis) was exchanged into 50 mM Tris-HCl, 100 mM NaCl (pH 7.5) buffer and concentrated for activity analyses.

### Co-substrate turnover experiments

Purified recombinant ALKBH5 was tested for its ability to stimulate ALKBH5-dependent decarboxylation of 1-[^14^C]-labelled 2OG, as described [38], [39]. Standard assay conditions comprised a total volume of 100 µl: 50 mM Tris·HCl pH 7.5, 4 mM ascorbate, 288 µM 2OG, 3.7 µM 1-[^14^C]-2OG (specific activity 56.8 µCi·nmol^−1^, stock concentration 1.83mM, Perkin Elmer NEN), 100 µM FeSO_4_·7H_2_O, 0.66 mg·ml^−1^ catalase, 4 µM enzyme. Briefly, the assay was set up in three drops, one containing enzyme (10 µl 40 µM), another containing buffer, substrate or inhibitor (10 µl 1 mM) and the third (80 µl) containing all other reagents prior to mixing in a 5 ml tube. A 0.5 ml Eppendorf vial containing 200 µl hyamine hydroxide was added and the tube was then sealed with a rubber septum. The reaction was started by mixing and incubated with shaking at 37°C for 15 minutes and then quenched with methanol (200 µl). Reaction tubes were then kept on ice for 20 minutes, before the hyamine hydroxide was removed and treated with scintillant liquid for radioactive count measurement in dpm. Conversion of 2OG was calculated from the percentage of 1-[^14^C]-2OG that had been converted to ^14^CO_2_ gas. Assays were performed in triplicate. For testing of potential substrates, the assay was performed as above, except that mixtures comprised three drops, one containing enzyme (10 µl 40 µM), another containing oligonucleotide (15 µl 100 µM) and the third (75 µl) containing all other reagents. The oligonucleotide sequence used was 5′-GCXAGGTCCCGTAGTGCG-3′, where X denotes the modified base. Oligonucleotides were purchased from ATDBio Ltd. (Southampton, UK) or Sigma-Aldrich and purified by desalting or reversed-phase HPLC as required. Oligonucleotide purity was checked by MALDI or HPLC analysis.

### LCMS-based oligonucleotide turnover experiments

Potential conversion of single-stranded DNA oligonucleotides by purified recombinant ALKBH5 was investigated using oligonucleotide LCMS analysis based on ion pairing. Incubations contained 5 µM enzyme, 10 µM oligonucleotide substrate, 50 µM FeSO_4_.7H_2_O, 300 µM 2OG, 4 mM sodium ascorbate and, optionally, 300 µM pyridine-2,4-dicarboxylic acid in a total volume of 50 µl with 50 mM Tris pH 7.5. Samples were incubated overnight at 37°C with shaking. The reactions were stopped by addition of 100 µl CH_3_CN, incubated on ice for 1 hour and centrifuged at 14,000 rpm for 10 minutes at 4°C. The supernatant was lyophilised and reconstituted in ultrapure water. Samples (10 µl) were run on a 2.5 µm Waters XBridge OST C18 Column (4.6×50 mm) at 0.5 ml/min. Elution employed a linear gradient from 10% to 70% buffer B in buffer A over 16 minutes. Buffers A and B contained 5% and 60% (v/v) methanol, respectively, in 400 mM hexafluoroisopropanol, 16.3 mM triethylamine, pH 8.0. Detection was performed on a Waters LCT Premier XE (Micromass) electrospray ionisation mass spectrometer connected to the HPLC system (HP1050 Series) in negative ion mode.

## Supporting Information

Figure S1 ***In silico* analysis of the ALKBH5 promoter.** Analysis of the *ALKBH5* promoter and conservation of identified putative transcription factor binding sites. Both identified putative HIF binding sites are highly conserved.
(TIF)

Figure S2 **No apparent activity of recombinant ALKBH5 on methylated oligonucleotides.** Single-stranded DNA oligonucleotides containing 1-methyladenine (1meA), 3-methylcytosine (3meC), 5-methylcytosine (5meC), 1-methylguanine (1meG) and 3-methylthymine (3meT) were incubated overnight with recombinant ALKBH5 and oligonucleotides were analyzed by ion-pairing LCMS. No modification is observed on any of the oligonucleotides.
(TIF)

Figure S3 **No apparent role for ALKBH5 in the DNA damage response.** U2OS cells treated with either scrambled or ALKBH5 siRNA were exposed to the periods of hypoxia and reoxygenation indicated and stained for either γH2AX (A) or 53BP1 (**B**). In each case the number of cells with >10 nuclear foci were scored. In each condition a minimum of 100 cells were scored. Doxorubicin (2 µM) was used a positive control for DNA damage induction. (**C**) U2OS cells were treated with scrambled (control) or ALKBH5 siRNA and exposed to 0.02% O_2_ for the times indicated. Cells were then returned to normal incubation conditions and colonies allowed to form. (**D**) U2OS cells were treated with scrambled (control) or ALKBH5 siRNA and exposed to 2.0% O_2_ for 24 hours. 30 minutes prior to reoxygenation cells were irradiated as indicated. Cells were then allowed to form colonies and counted.
(JPG)
