# Supplementary figures and images for: Human AlkB Homologue 5 Is a Nuclear 2-Oxoglutarate Dependent Oxygenase and a Direct Target of Hypoxia-Inducible Factor 1α (HIF-1α)
Source: PLoS One. 2011 Jan 14;6(1):e16210. doi: 10.1371/journal.pone.0016210 (PMC3021549; doi:10.1371/journal.pone.0016210)

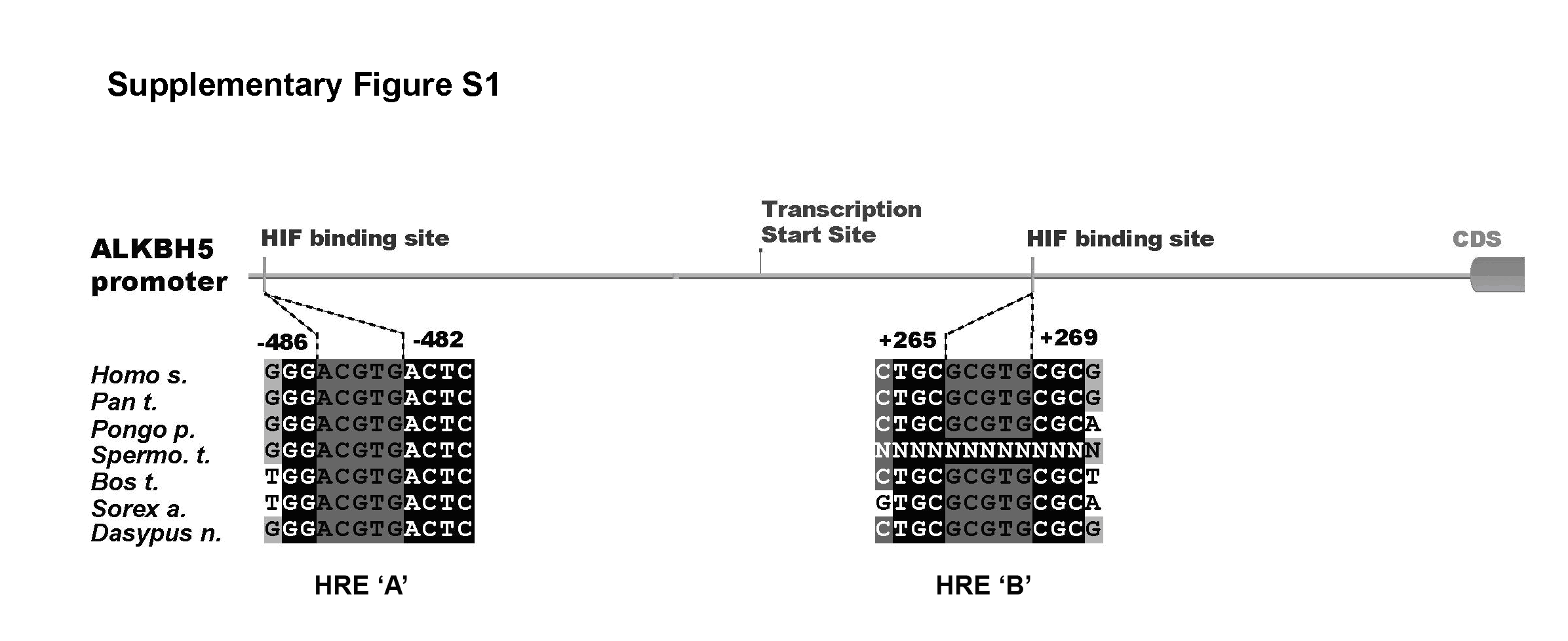

Supplement: Figure S1 — In silico analysis of the ALKBH5 promoter. Analysis of the ALKBH5 promoter and conservation of identified putative transcription factor binding sites. Both identified putative HIF binding sites are highly conserved. (TIF) [file pone.0016210.s001.tif]

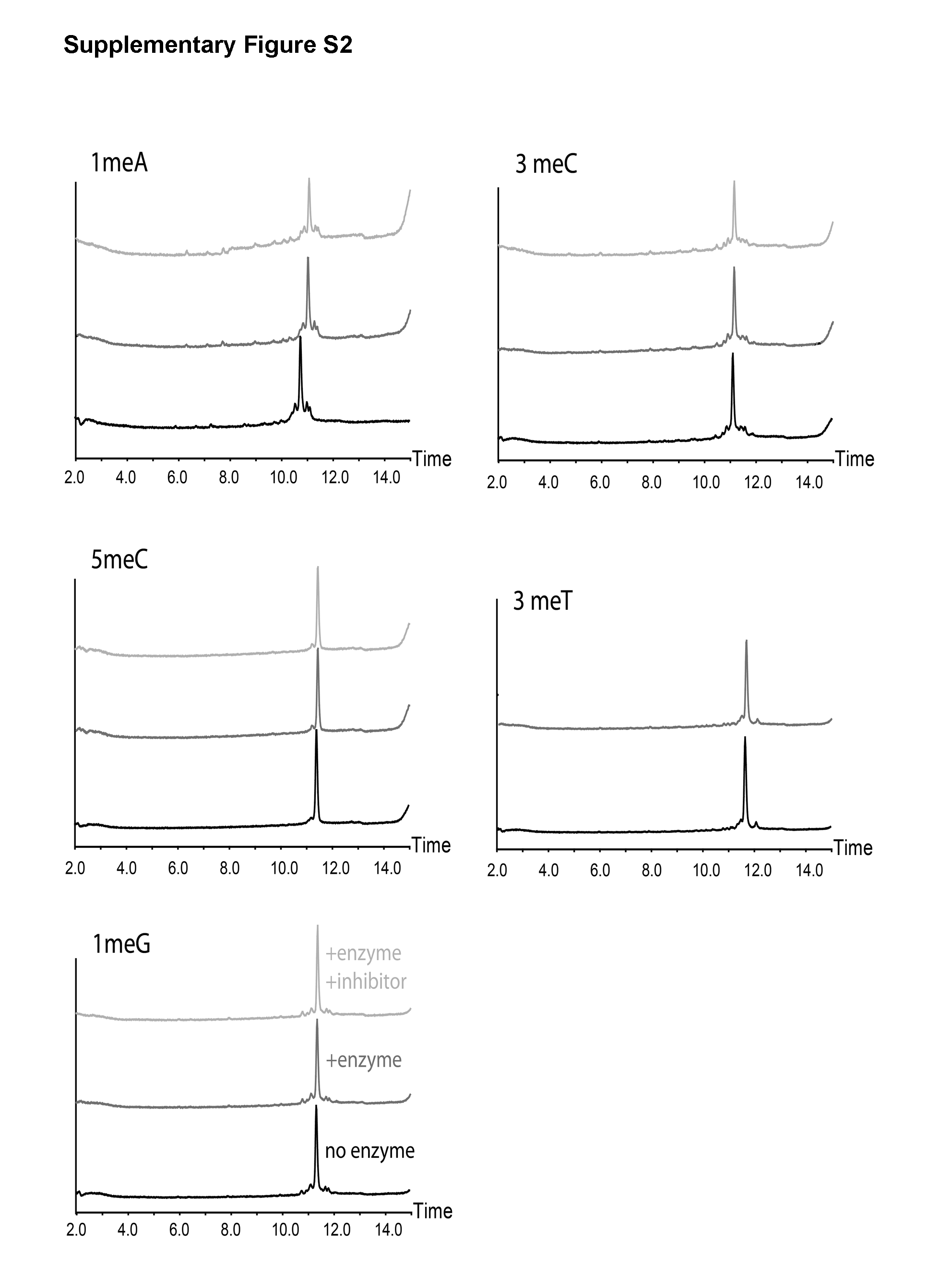

Supplement: Figure S2 — No apparent activity of recombinant ALKBH5 on methylated oligonucleotides. Single-stranded DNA oligonucleotides containing 1-methyladenine (1meA), 3-methylcytosine (3meC), 5-methylcytosine (5meC), 1-methylguanine (1meG) and 3-methylthymine (3meT) were incubated overnight with recombinant ALKBH5 and oligonucleotides were analyzed by ion-pairing LCMS. No modification is observed on any of the oligonucleotides. (TIF) [file pone.0016210.s002.tif]

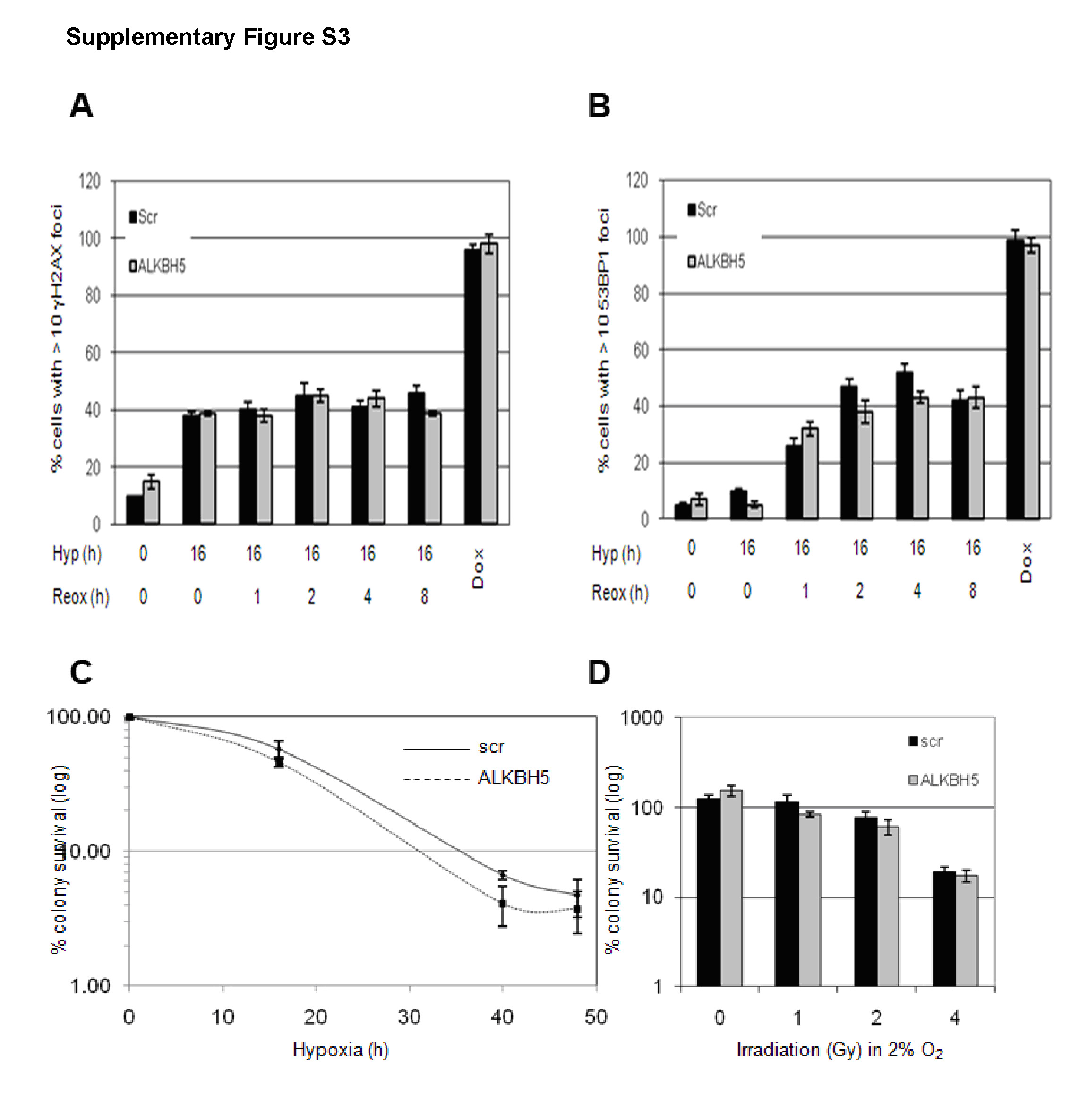

Supplement: Figure S3 — No apparent role for ALKBH5 in the DNA damage response. U2OS cells treated with either scrambled or ALKBH5 siRNA were exposed to the periods of hypoxia and reoxygenation indicated and stained for either γH2AX (A) or 53BP1 (B). In each case the number of cells with >10 nuclear foci were scored. In each condition a minimum of 100 cells were scored. Doxorubicin (2 µM) was used a positive control for DNA damage induction. (C) U2OS cells were treated with scrambled (control) or ALKBH5 siRNA and exposed to 0.02% O2 for the times indicated. Cells were then returned to normal incubation conditions and colonies allowed to form. (D) U2OS cells were treated with scrambled (control) or ALKBH5 siRNA and exposed to 2.0% O2 for 24 hours. 30 minutes prior to reoxygenation cells were irradiated as indicated. Cells were then allowed to form colonies and counted. (JPG) [file pone.0016210.s003.jpg]
